# Pharmacological mechanism of mylabris in the treatment of leukemia based on bioinformatics and systematic pharmacology

**DOI:** 10.1080/21655979.2021.1943110

**Published:** 2021-07-05

**Authors:** Huali Zhan, Yujiao Bai, Yu Lv, Xianqin Zhang, Lin Zhang, Shanshan Deng

**Affiliations:** a Department of Humanities and Social Sciences, Zhejiang Industry Polytechnic College, Zhejiang, China; b Non-Coding RNA and Drug Discovery Key Laboratory of Sichuan Province, Chengdu Medical College, Chengdu, Sichuan, China; c School of Basic Medical Sciences, Chengdu Medical College, Chengdu, Sichuan, China; d Wenzhou Medical University Renji College, Wenzhou, Zhejiang, China; e College of Pharmaceutical Sciences, Zhejiang Chinese Medical University, Hangzhou, Zhejiang, China; f Department of Pharmacy, Shaoxing People’s Hospital; Shaoxing Hospital, Zhejiang University School of Medicine, Shaoxing, Zhejiang, China

**Keywords:** Mylabris, leukemia, bioinformatics, systematic pharmacology, p53 signaling

## Abstract

Leukemia is a common blood cancer, whose treatment usually necessitates chemo/radiotherapy and bone marrow transplant. Hence, safer and more effective options are urgently needed. Mylabris, the dried body of blister beetles, has been used extensively in traditional Chinese medicine. This study applied bioinformatics and systematic pharmacology to investigate the mechanism of action of mylabris in the treatment of leukemia. Five effective components and 35 corresponding target proteins were identified by screening the TCMSP database; whereas 776 genes related to leukemia were selected using OMIM, GeneCards, and the Therapeutic Target Database. Eight genes common to mylabris and leukemia were identified. Protein-protein interaction network analysis and a component-target-pathway diagram identified *TP53* and *PTEN* as key gene targets of mylabris in the treatment of leukemia. GO enrichment analysis pointed to DNA damage and cell cycle disorder caused by p53 signaling as the most significant processes; whereas KEGG enrichment pointed to the p53 signaling pathway. In summary, mylabris may exert a therapeutic effect on leukemia by triggering DNA damage, inducing apoptosis, as well as inhibiting the growth and proliferation of tumor cells through the regulation of *TP53* and *PTEN*. These findings provide a mechanistic rationale for the treatment of leukemia with traditional Chinese medicine.

## Graphical abstract

**Figure:**
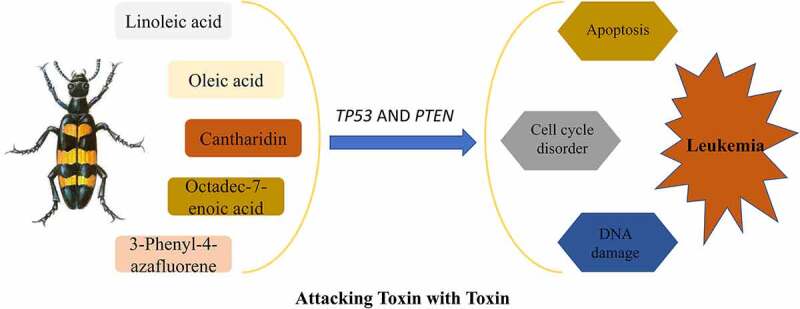

## 1. Introduction

Leukemia is a malignant clonal disease of hematopoietic stem cells. Based on cell type, it can be classified into myeloid, lymphocytic, and mixed-cell leukemia. The degree of cell differentiation and duration of the disease is used to further discriminate between acute leukemia and chronic leukemia [1]. Leukemia has been found to be directly related to viral infection, radiation, and chemical or drug toxicity. Traditional Chinese medicine classifies leukemia as ‘syndrome accumulation’, ‘blood deficiency’, and ‘blood syndrome’. These characteristics are due mainly to the lack of vital qi in the body, which leads to liver and spleen deficiency, colonization by poisonous pathogens, blood-heat, and other diseases. At present, leukemia is treated chiefly by chemotherapy, radiotherapy, targeted therapy, immunotherapy, and stem cell transplantation. Combined traditional Chinese medicine can increase chemotherapy sensitivity, reduce drug resistance of cancer cells, prolong patient survival, and prevent recurrence of the disease [2,3]. In recent years, traditional Chinese medicine has come to encompass the idea that exogenous ‘toxins’ of natural, radioactive or synthetic origin are the causes of leukemia, because they invade and injure the bone marrow. While arsenic trioxide has been successfully applied in the treatment of acute promyelocytic leukemia [4], addition of toxic substances from the beetle *Mylabris centipede* or the herb *Hedyotis diffusa* is believed to further improve leukemia treatment [4]. Hence, the traditional Chinese medicine tenet of ‘attacking toxin with toxin’ has emerged as a potential therapeutic approach for leukemia.

First recorded in ‘Shennong’s Herbal Classics’, the mylabris, or dried body of the southern large or yellow-black *Mylabris* blister beetles of *Daphne genkwa*, is effective at disrupting blood stasis, dispersing knots, overcoming dysentery, and attacking noxious and erosive sores [5]. Cantharidin, whose chemical name is hexahydro-3α, 7α-dimethyl-4,7-epoxyisobenzofuran-1,3-dione, is the main medicinal component of *Mylabris* beetles and mylabris extract [6]. Cantharidin has a good anti-tumor effect, which was first reported in 1980 by Chen et al [7]. In recent years, an improved understanding of the anti-tumor function of various traditional Chinese treatments has revealed that cantharidin exerts a specific therapeutic effect on malignant and advanced tumors. Cantharidin has been documented to promote apoptosis and alter protein synthesis by inhibiting protein phosphatase 1 (PP1) and protein phosphatase 2A (PP2A) [8]. Cantharidin can inhibit the proliferation of leukemic cells, induce apoptosis, block the cell cycle, and enhance the inhibitory effect of chemotherapeutic drugs [9–11]. In addition, it can delay tumor progression, invasion, and metastasis [12]. At present, the therapeutic mechanism employed by mylabris against tumors remains unknown.

This study aims to use bioinformatics and systematic pharmacology to explore the targets and signal transduction pathways triggered by mylabris in the treatment of leukemia. The study also aims to provide preliminary data on the possible mechanism of action of mylabris and, therefore, its clinical application in the treatment of leukemia. An improved scientific understanding of the molecular mechanism of mylabris and its derivatives in the treatment of leukemia will contribute to the modernization of traditional Chinese medicine.

## 2. Materials and methods

### 2.1. Selection and target screening of mylabris active components

The traditional Chinese medicine system pharmacology (TCMSP) database (https://tcmspw.com/tcmsp.php) contains information about the active components, bioavailability, drug-like property, and other parameters of Chinese herbal medicines [13]. ‘Mylabris’ was used as the search term in the TCMSP database to find out its effective active ingredients. The search conditions were bioavailability ≥ 30% and drug-like property ≥ 0.10. The effective active ingredients identified by the screening were then matched with their putative target proteins based on molecular weight.

### 2.2. Correspondence between effective components and potential targets

The target proteins corresponding to mylabris active components were imported into the UniProt database (www.uniprot.org). The screening conditions were set to ‘Human’ and the corresponding genes were identified [14]. The active ingredients and potential targets of mylabris were imported into Cytoscape 3.7.2 (https://cytoscape.org) for visualization, and an active ingredients-target interaction network diagram was obtained.

### 2.3. Collection and collation of disease genes

Leukemia-related genes were collated using the keyword ‘Leukemia’ in Online Mendelian Inheritance in Man (OMIM) (http://www.omim.org/), Therapeutic Target Database (TTD) (http://db.idrblab.net/ttd/), PharmGKB (https://www.pharmgkb.org/), and GeneCards (https://www.genecards.org) databases [14]. The Hiplot visualization mapping website (https://hiplot.com.cn) was used to construct an intersection network of disease genes obtained from the various databases.

### 2.4. Determination of common targets of drugs and disease genes

To obtain common effective component gene targets, the intersection of active ingredient gene targets and leukemia-related gene targets of mylabris was drawn by Venny (https://bioinfogp.cnp.csic.es/tools/venny/).

### 2.5. Construction of a protein interaction network

The search tool for the retrieval of interacting genes (STRING) database (https://string-db.org/) can be used to predict correlations between proteins. By visualizing the strength and connection of a protein-protein interaction (PPI), the close relationship between proteins can be scored. The higher is the score, the higher is the confidence of the PPI [15]. The common gene targets of mylabris active components and leukemia-related genes were processed by the STRING database. The conditions were set to ‘Multiple proteins’ and ‘Homo sapiens’ to obtain the protein interaction network map, and a high confidence level (0.900) was set to improve the accuracy of the data. The common gene targets were imported into Cytoscape 3.7.2 for data processing. Key genes were defined as those with degree value > average value.

### 2.6. Functional enrichment analysis

Gene Ontology (GO) [16] and Kyoto Encyclopedia of Genes and Genomes (KEGG) [17] enrichment analyses were carried out using all intersecting genes because the common gene targets of active ingredients and leukemia-related disease genes were not sufficient. The potential targets of mylabris for the treatment of leukemia were imported in the Metascape database (http://metascape.org). GO enrichment analysis included molecular function, biological pathways, and cellular components, and was performed using a web-based bioinformatics tool (http://www.bioinformatics.com.cn/). KEGG is a useful resource for systematic analysis of gene function and related advanced gene information. KEGG enrichment results were visualized in Hiplot.

### 2.7. Construction of a component-target-pathway diagram

The main components and related intersecting targets of mylabris in the treatment of leukemia were found by KEGG enrichment analysis. An effective components-target-pathway diagram was constructed in Cytoscape 3.7.2 [17].

## 3. Results

Mylabris, a traditional Chinese remedy consisting of the dry body of bristle beetles, has been shown to have anti-tumor effect, yet its mechanism of action against leukemia is not known. This study applied bioinformatics and systematic pharmacology to investigate the molecular mechanism of mylabris in the treatment of leukemia.Based on the bioinformatics identification of common targets of mylabris and leukemia-related genes, we show that the effective components of mylabris act simultaneously on two key gene targets, PTEN and TP53, as well as the p53 signaling pathway, to inhibit the proliferation of leukemic cells. Hence, mylabris exerts a therapeutic effect on leukemia through its synergistic action on multiple compounds, targets, and signaling pathways.

### 3.1. Drug-target interaction

**Figure 1.**
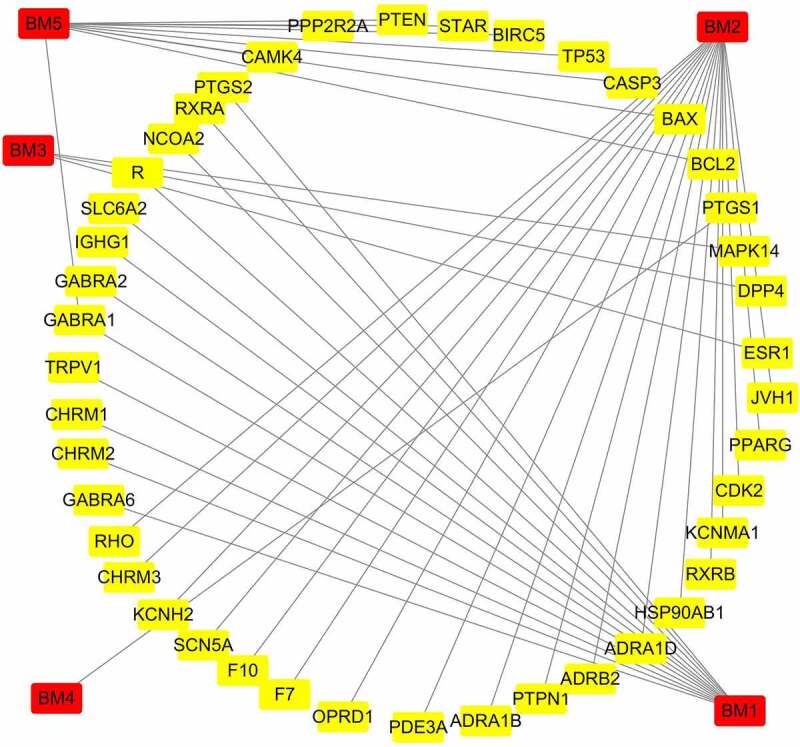
Interaction diagram between active ingredients and potential targets

Screening of the TCMSP database yielded a total of five active components of mylabris, which are listed in Table 1. Based on molecular weight, these components were matched with 35 target proteins. As shown in Figure 1, the relationship between the identified active components and 35 targets is characterized by a total of 48 nodes and 44 edges.

**Table 1.** The effective components of Mylabris screened using TCMSP database

No	Name	Compound	OB (%)	DL
**1**	BM1	linoleic acid	41.90	0.14
**2**	BM2	oleic acid	33.13	0.14
**3**	BM3	3-Phenyl-4-azafluorene	32.90	0.23
**4**	BM4	octadec-7-enoic acid	33.13	0.14
**5**	BM5	Cantharidin	51.23	0.10

OB, bioavailability; DL, drug-like property.

Red circle indicates active ingredients, yellow box indicates target.

### 3.2. Prediction of mylabris target genes and leukemia disease genes

**Figure 2.**
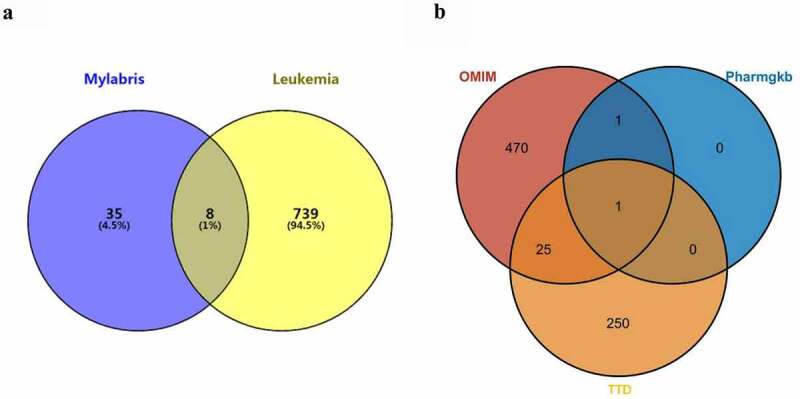
Intersection of mylabris target genes and leukemia disease genes

As there was no intersection between the leukemia-related genes in the GeneCards database and the compounds in the TCMSP database, the query results of the GeneCards database were omitted. A total of 776 leukemia-related genes were identified, including 497 in the OMIM database, 2 in the PharmGKB database, and 277 in the TTD database. Eight gene targets common to mylabris effective components and leukemia were obtained (Figure 2(a)). One leukemia-related gene, *MTHFR*, encoding methylene tetrahydrofolate reductase, was common to all three databases (PharmGKB, TTD, and OMIM); whereas 26 genes were common to the OMIM and TTD databases (Figure 2(b)).

Figure (a) indicates the intersection states of mylabris effective components and leukemia-related genes. Figure (b) indicates the intersection leukemia disease genes obtained from Pharmgkb, TTD and OMIM databases.

### 3.3. Analysis of the PPI network related to leukemia

**Figure 3.**
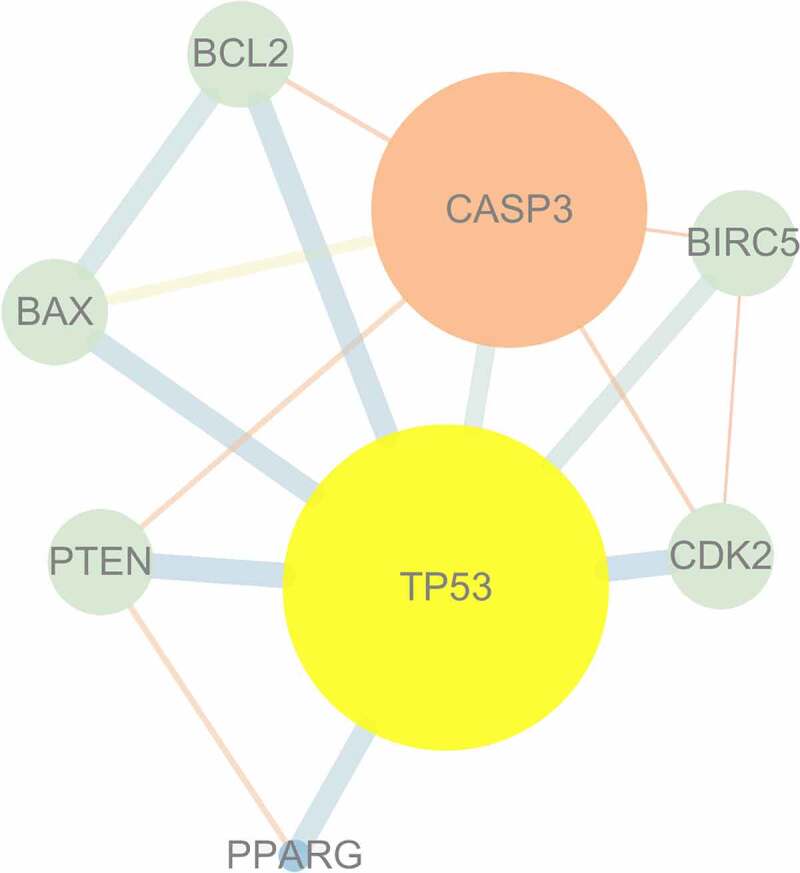
PPI network interaction among intersection targets

The PPI network was generated using the targets shared by mylabris active ingredients and leukemia-related genes. The relationship between potential gene targets revealed 8 nodes and 15 edges, as shown in Figure 3. *TP53*, encoding the tumor protein p53, and *CASP3*, encoding caspase 3, were identified as key genes because their degree value was greater than the average value of 3.75. *TP53* exhibited the highest number of connections and, hence, closest relationship with other potential therapeutic gene targets, supporting its central role in the treatment of leukemia.

The larger the circle is, the larger the degree value is; the redder the color is, the more important the node is; and the thicker the line between the nodes is, the stronger the interaction is.

### 3.4. GO and KEGG enrichment analyses

#### 3.4.1. GO enrichment analysis

**Figure 4.**
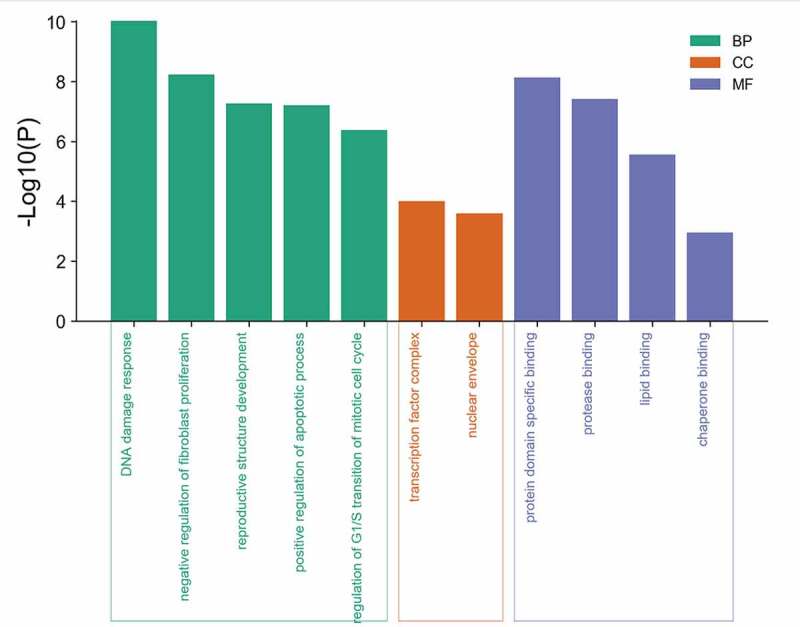
GO enrichment analysis of 8 potential targets

GO enrichment analysis of eight potential genes common to mylabris effective components and leukemia was carried out using the Metascape database. Five biological processes, two molecular components, and four molecular functions were obtained (Figure 4). GO analysis revealed that the active components of mylabris could alter the cell cycle of leukemic cells through p53 signaling, cause DNA damage, affect the inhibition of proliferating fibroblasts, positively regulate apoptosis in leukemic cells, and negatively regulate the cell cycle G1/S transition. The active components of mylabris acted not only on transcription factor complexes and the nuclear membrane, but affected also the GO functions of leukemic cells, including protein domain, protease, and lipid binding. Given that p53 signaling yielded the strongest GO enrichment result and DNA damage was also among the hits together with cell cycle disorder, we believe that the effective components of mylabris lead to cell cycle arrest in leukemic cells through p53-dependent signal transduction, a critical mediator of DNA damage.

Green corresponds to biological process (BP), orange corresponds to cellular composition (CC), and blue corresponds to molecular function (MF). The X axis represents the significance of enrichment, the larger the value, the more obvious, and the Y axis represents the gene term. P < 0.001 indicates significant enrichment.

#### 3.4.2. KEGG enrichment analysis

**Figure 5.**
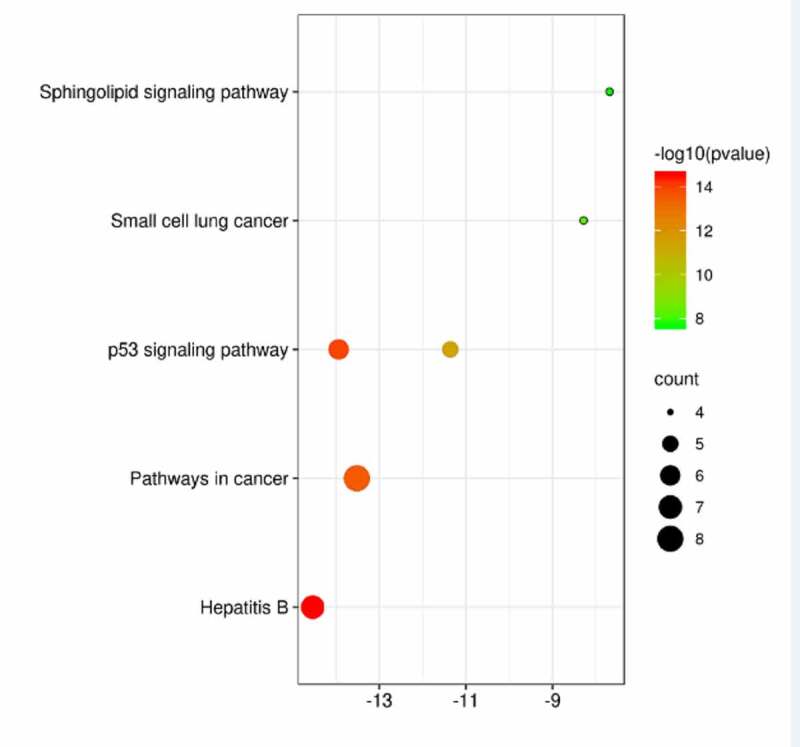
KEGG pathway enrichment analysis of potential acting genes

KEGG enrichment analysis of eight potential genes common to the active ingredient genes of mylabris and leukemia yielded six signaling pathways with P < 0.001 (Figure 5). They included the ‘P53 signaling pathway’, which appeared twice, as well as ‘Hepatitis B’, ‘Pathways in cancer’, ‘Small cell lung cancer’, and ‘Sphingolipid signaling pathway’.

The Y axis represents the corresponding pathway, the X axis represents the significance of enrichment, the larger the more significant, the redder the color, the more obvious the enrichment, and the size of the ball represents the amount of genes contained. P< 0.001 indicates significant enrichment

### 3.5. Component-target-pathway diagram

**Figure 6.**
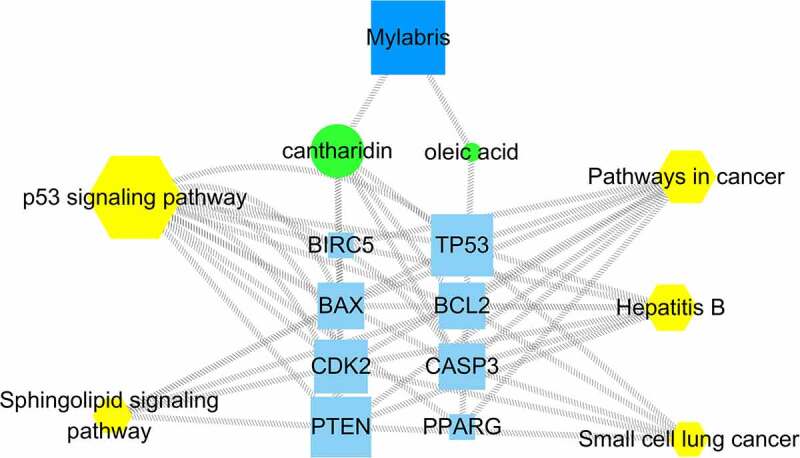
Active ingredient-target-path diagram

Mylabris might exert its therapeutic effect on leukemia through multiple components, pathways, and targets. Importantly, each target and pathway appeared to be interrelated and to interact with others. Five pathways enriched in KEGG analysis were mapped against cantharidin and oleic acid, two mylabris components effective against leukemia, to generate the effective components-target-pathway diagram (Figure 6). The diagram contained 54 nodes and 101 edges. The centrality and degree values were highest for *TP53* and phosphatase and tensin homolog (*PTEN*). Accordingly, *TP53* and *PTEN* may be the key genes for the treatment of leukemia. In addition, the degree value of the p53 pathway was significantly higher than that of other signaling pathways, suggesting that p53 might be the best signaling pathway for the treatment of leukemia.

Green spherical nodes represent the active components of mylabris, blue spherical nodes represent mylabris, blue square nodes represent effective targets, and yellow heptagonal nodes represent signal pathways.

## 4. Discussion

Leukemia includes a group of life-threatening malignant disorders of the blood and bone marrow [18]. The disease progresses rapidly and leads to numerous complications. Various therapeutic approaches exist, and the treatment plan needs to be formulated in combination with detailed clinical classification and prognosis stratification. At present, chemotherapy and bone marrow transplantation remain the treatments of choice. However, due to the considerable side effects of chemotherapy and elevated likelihood of recurrence, the therapeutic effect is not optimal, especially for patients with acute leukemia [19]. For its part, bone marrow transplantation is a double-edged sword, as it can lead to low immunity of patients and postoperative infection. Although the etiology of leukemia is becoming increasingly known, its exact pathogenesis remains poorly understood. Hence, safer and more effective drugs, with fewer side effects need to be developed. Ongoing advances in clinical research have brought further attention to the treatment of leukemia with Chinese herbal medicine and its extracts.

A large number of studies have confirmed the strong anticancer potential of mylabris treatment and its successful application against various cancers. Cantharidin is the main bioactive component of mylabris. So far, it has been used in the treatment of liver cancer, pancreatic cancer, and bladder cancer. As early as 1989, mylabris was shown to possess anti-tumor properties, such as stimulating bone marrow cells and increasing the number of white blood cells [6]. Lixia et al [20]. used anti-human CD19 monoclonal antibody secreted by ZCH-4-2E8 cells to ligate norcantharidin (NCTD), and successfully synthesized the immunotoxin 2E8-NCTD. The results showed that the immunotoxin had an excellent targeting killing effect on CD19+ Nalm-6 leukemia cells in vitro. Ruirong et al [21]. found that cantharidin significantly inhibited the proliferation of leukemia HL60 cells, and this inhibitory effect might be related to the down-regulation of telomerase reverse transcriptase (hTERT) mRNA expression. Recent studies have shown that *NUR77*, which encodes nuclear receptor subfamily 4 group A member 1, plays an important role in tumor suppression in acute myeloid leukemia. Specifically, Zanyang et al [22]. reported that cantharidin induced *NUR77* expression, significantly inhibiting viability and colony formation in acute myeloid leukemia cell lines. The above evidence clearly supports the potential of using mylabris for the treatment of leukemia; however, its specific cytotoxicity mechanism remains to be determined.

Increasingly, effective gene targets are identified by combining simulation, high-throughput omics analysis, and big data processing and mining. This synergistic approach offers a new strategy for determining the efficacy and pharmacological mechanism of Chinese medicine and its derivatives in the treatment of diseases. Using bioinformatics and systematic pharmacology, we first screened the TCMSP database for mylabris components effective against leukemia, which yielded cantharidin, oleic acid, linoleic acid, octadecane-7-enoic acid, and 3-phenyl-4-azafluene (Table 1). Consistent with previous studies, cantharidin was the main effective component of mylabris. The renown anti-cancer effect of cantharidin relies on: 1) inhibiting the growth and proliferation of cancer cells by triggering DNA damage and down-regulating BCR-ABL transcription; 2) inducing apoptosis of cancer cells through intrinsic or extrinsic apoptotic pathways; 3) inducing DNA damage and inhibiting expression of proteins involved in damage repair; 4) inducing cell cycle arrest; 5) blocking relevant signaling pathways; 6) regulating the expression of metastasis-related proteins and inhibiting cancer cell metastasis; and 7) regulating autophagy-related genes to induce autophagy in cancer cells [12]. Thus, understanding the mechanism of action of cantharidin could help expand its use as an anti-tumor agent.

*MTHFR* is a key gene responsible for regulating glial metabolism and DNA methylation [23]. Moreover, MTHFR is the key enzyme in folic acid metabolism [24]. Two common genetic polymorphisms of *MTHFR*, A1298C and C677T, can lead to decreased MTHFR enzyme activity [25]. This phenomenon is related to abnormal glial metabolism and DNA hypomethylation [26], both of which have been associated with the development of leukemia [25]. Several studies have investigated the relationship between *MTHFR* polymorphism and leukemia, with some similarities and differences among the results. On the one hand, *MTHFR* polymorphism appears to be strongly associated with the occurrence and development of leukemia [24–28]. On the other hand, Aly et al [25]. reported a correlation between *MTHFR* C677T mutation and chronic myelogenous leukemia, while other studies could not confirm a significant relationship between the C677T mutation and the disease [29,30]. In our study, *MTHFR* was the only gene common to all three queried databases, i.e., PharmGKB, TTD, and OMIM. Our results establish a preliminary correlation between *MTHFR* and leukemia. However, whether *MTHFR* polymorphism actually causes leukemia or affects its development will require further studies.

*TP53* is the most commonly mutated gene among known human cancer genes. It acts as a transcription factor and is widely expressed in cells. P53 regulates a series of physiological activities including aging, development, and cell metabolism. Disruption of the p53 pathway is a common feature of many malignant tumors [31]. *TP53* and the p53 signaling pathway are central targets in the treatment of leukemia. Numerous studies have shown that inactivation of *TP53* can lead to acute myeloid leukemia, which makes *TP53* both a challenge and an opportunity for the treatment of this disease [32]. *TP53* is the key target of cantharidin, and inhibits also PP1 and PP2A. The 13B regulatory subunit of PP1 (PP1R13B) can cooperate with *TP53* to promote apoptosis of tumor cells [33]. PP1 and PP2A, the key enzymes regulating the cell cycle, control also other cell processes, including signal transduction, glucose metabolism, and calcium transport [34]. Cantharidin is related to signal transduction during the cell cycle, mitosis, and apoptosis, accelerating entry into S phase and regulating apoptosis in G2/M phase. It is mainly active during the M phase of cell growth and reproduction, with additional minor activity during interphase [35]. Efferth et al [34]. reported that cantharidin induced apoptosis of leukemia cells, resulting in DNA single-strand and double-strand breaks. Consistent with previous studies, the present GO analysis revealed that mylabris could regulate cell apoptosis, alter the division cycle, and affect transcription factors, thus impacting the normal replication and expression of DNA. P53-mediated signal transduction leads to cell cycle disorders and DNA damage. Here, the p53 signaling pathway appeared to be the most important mediator in the treatment of leukemia using mylabris. Future experiments should verify the predicted targets and pathways.

The role of PTEN in the treatment of leukemia by mylabris should not be underestimated, either alone or in combination with p53. PTEN is a double phosphatase with both protein phosphatase and lipid phosphatase activity. In 1997, it was independently characterized as a tumor suppressor by three different laboratories [36–38]. It was later proved that PTEN was the main negative regulator of the cell growth and survival signaling pathway, and played an important role in cell metabolism [39,40]. PTEN is present in the cytoplasm and nucleus. Nuclear PTEN can stabilize chromosomes, participate in DNA repair, and regulate the cell cycle [40]. There is a close relationship between PTEN and p53, with PTEN being directly linked to p53 in the nucleus. Stability and transcriptional activity of the tumor suppressor *TP53* are significantly enhanced by phosphatase-dependent or non-phosphatase-dependent mechanisms [41]. Whether PTEN and p53, the two key targets of mylabris in the treatment of leukemia, play a synergistic role will be determined by future studies.

## 5. Conclusions

In summary, this paper studies the molecular mechanism of mylabris in the treatment of leukemia based on bioinformatics and systematic pharmacology. We found that a variety of compounds in mylabris can play a role in the treatment of leukemia through the synergistic effect of targets and signal pathways. The active components of mylabris can act on both PTEN and TP53, the key gene targets, and exert pharmacological effects through p53 signal pathway. In this way, it can block the cell cycle, induce apoptosis, and eventually lead to DNA damage and inhibit the proliferation of leukemic cells.

## Data Availability

All of the datasets analyzed were acquired from the traditional Chinese medicine system pharmacology (TCMSP) database (https://tcmspw.com/tcmsp.php), Online Mendelian Inheritance in Man (OMIM) database (http://www.omim.org/), Therapeutic Target Database (TTD) database (http://www.db.idrblab.net/ttd/), PharmGKB database (https://www.pharmgkb.org/) and GeneCards database (https://www.genecards.org).

## Highlights

Traditional Chinese medicine’s approach posits to ‘attack toxicity by toxicity’.

The *Mylabris* toxin has been applied in this bioinformatics study to treat leukemia.

Mylabris acts on multiple components, targets, and pathways driving leukemia.

*TP53* and *PTEN* as well as p53 signaling are key elements targeted by mylabris.

## Abbreviations

PP1: protein phosphatase 1; PP2A: protein phosphatase 2A; TCMSP: traditional Chinese medicine system pharmacology; OB: oral bioavailability; DL: drug-like property; OMIM: Online Mendelian Inheritance in Man; TTD: Therapeutic Target Database; STRING: Search tool for the retrieval of interacting genes; PPI: protein-protein interaction; GO: gene ontology; KEGG: Kyoto Encyclopedia of Genes and Genomes; AML: acute myelocytic leukemia; PP1R13B: regulatory subunit 13B of PP1; PTEN: phosphatase and tensin homolog deleted on chromosome 10; CML: chronic myelogenous leukemia.
